# Hair EDX Analysis—A Promising Tool for Micronutrient Status Evaluation of Patients with IBD?

**DOI:** 10.3390/nu13082572

**Published:** 2021-07-27

**Authors:** Georgiana-Emmanuela Gîlcă-Blanariu, Adina Coroabă, Manuela Ciocoiu, Anca Trifan, Gabriel Dimofte, Smaranda Diaconescu, Vlad-Adrian Afrăsânie, Gheorghe G. Balan, Tudor Pinteală, Gabriela Ștefănescu

**Affiliations:** 1 Faculty of Medicine, Grigore T Popa University of Medicine and Pharmacy, 700115 Iași, Romania; georgiana.gilca@gmail.com (G.-E.G.-B.); mciocoiu2003@yahoo.com (M.C.); ancatrifan@yahoo.com (A.T.); gdimofte@gmail.com (G.D.); vlad_afrasanie@yahoo.com (V.-A.A.); balan.gheo@yahoo.com (G.G.B.); gabriela.stefanescu@gmail.com (G.Ș.); 2 Petru Poni Institute of Macromolecular Chemistry, 700487 Iași, Romania; adina.coroaba@icmpp.ro; 3 Sf Spiridon County Clinical Emergency Hospital, 700111 Iași, Romania; tudor.pinteala@gmail.com

**Keywords:** inflammatory bowel disease, micronutrients, EDX, trace element, iron, zinc, manganese, magnesium, calcium, copper

## Abstract

Micronutrient deficiencies can arise in various conditions, including inflammatory bowel diseases (IBD), and diagnosing these deficiencies can be challenging in the absence of specific clinical signs. The aim of this study was to evaluate the status of various trace elements hair concentration in IBD patients compared to a healthy control group and to identify potential correlations between the micronutrient status and relevant parameters related to disease activity. The concentrations of iron, magnesium, calcium, zinc, copper, manganese, selenium and sulfur in the hair of 37 IBD patients with prior diagnosed IBD (12 Crohn’s disease and 25 ulcerative colitis) and 31 healthy controls were evaluated by Energy Dispersive X-Ray spectroscopy (EDX). Significant differences in hair concentration profile of studied trace elements were identified for IBD patients compared to healthy controls. A significantly decreased hair concentration of iron, magnesium, calcium and selenium and a significantly increased sulfur hair concentration were observed in IBD patients at the time of evaluation. A decreased hair calcium concentration (*r* = −0.772, *p* = 0.003) and an increased sulfur concentration (*r* = 0.585, *p* = 0.046) were significantly correlated with disease activity. Conclusion: Hair mineral and trace elements evaluation may contribute to a proper evaluation of their status in IBD patients and improving the management of nutritional status of IBD patients.

## 1. Introduction

Inflammatory bowel disease (IBD) represents chronic inflammatory conditions of the gastrointestinal tract, characterized by periods of disease flares alternating with remission. Genetic predisposition, environmental factors and dysbiosis are significant factors involved in IBD pathogenesis [1,2,3].

Malnutrition, including protein-calorie malnutrition or micronutrient deficiency, is common among IBD patients and may persist throughout the course of the disease because of altered intake, impaired absorption, direct gastrointestinal loss or hypercatabolic state [4]. Additionally, nutritional deficiencies are associated with a significant risk of poor outcomes, in terms of prolonged hospitalization [5,6], complicated perioperative course and higher mortality [7]. Because inflammation might affect serum micronutrient concentrations, serum levels can have a limited value in reflecting the status of some nutrients in chronic inflammatory diseases such as IBD [4]. Our aim was to investigate the usefulness of measuring the hair concentration of mineral and trace elements in IBD patients, compare them to healthy controls and investigate potential correlations between the micronutrient status and the relevant parameters related to disease activity.

## 2. Materials and Methods

### 2.1. Subjects

We performed an observational study, including patients diagnosed with ulcerative colitis (UC) (*n* = 25) and Crohn’s disease (CD) (*n* = 17). The inclusion criteria for the study group were: confirmed diagnosis of IBD based on the clinical presentation as well as endoscopic and histopathological findings, age between 18 and 70 years and written consent of the patients. Patients with overlapping infections, using micronutrients supplements, with a history of neoplasia or metabolic disorders (obesity, dyslipidemia and thyroid dysfunction) were excluded. Disease activity was evaluated by calculating the Crohn’s disease activity index (CDAI) for CD patients and a Mayo score for UC. The control group consisted of healthy subjects (*n* = 37), namely without history of chronic diseases, gastrointestinal disorders and autoimmune diseases. The use of hair therapeutic agents or cosmetics (medicinal shampoos which can interfere with the results of the investigations) and restrictive diets in the past 6 months represented exclusion criteria for both the study and the control group. Hair trace elements concentrations for iron (Fe%), magnesium (Mg%), calcium (Ca%), zinc (Zn%), copper (Cu%), manganese (Mn%), selenium (Se%) and sulfur (S%), serum albumin concentration, inflammatory markers, such as C reactive protein (CRP), and complete blood count, including calculating the neutrophil-to-lymphocyte ratio (NLR), were evaluated both for the study and control group. A colonoscopy was performed for all IBD patients and CT scans were also added when needed to assess disease extension.

### 2.2. Hair Sample Collection and SEM and EDX Measurement

The hair sample was collected from the occipital region and included a minimum of 10 strands for each patient. The EDX analyses were performed on the root end, after the samples were cleaned using the Hess procedure. The procedure consisted of placing the hair strands in small dishes with distilled water containing a drop of detergent and sonicating them for 5 min. The sample were afterwards washed in distilled water, sonicated for 5 min in absolute acetone and allowed to dry [8,9].

After the cleaning process, the hair samples were examined with a Quanta 200 Scanning Electron Microscope (SEM) at 30 kV in high vacuum mode. The EDX system mounted on the Quanta 200 SEM was used for element identification and performing the quantitative analysis. The elemental analysis was performed both on the surface and the cross section of the hair specimens. No secondary treatment or processing was necessary.

### 2.3. Ethical Considerations

The processing of hair samples was in accordance with the European Directive EC No 206 and the protocol used for processing hair samples undergoing EDX analysis was approved by the Scientific Council of Petru Poni Institute of Macromolecular Chemistry. The study protocol and all procedures included in the study were in accordance with the ethical standards within the Declaration of Helsinki and approved by the Ethical Committee of Grigore T Popa University of Medicine and Pharmacy (25 November 2018), Sf Spiridon County Clinical Emergency Hospital (No 45/04.09/2019). Upon inclusion in the study, written informed consent was obtained from all patients enrolled.

### 2.4. Statistical Analysis

The statistical analysis was performed using SPSS v25.0 (SPSS, Inc, Chicago, IL, USA). Continuous variables were reported as mean values and standard deviation, or as median with 25th–75th percentiles. Qualitative variables were reported as absolute frequencies and relative frequencies (%). The comparisons between the analyzed groups were performed using a Student’s *t*-test, Kruskal–Wallis test or Chi-square test, depending on the homogeneity of the data series. For comparisons among three groups, a one-way ANOVA analysis was used. For evaluating various correlations of the studied parameters, a Pearson correlation or Spearman correlation test was performed, depending on the distribution of the data.

## 3. Results

### 3.1. Patient Characteristics

Our study included 37 patients with IBD (25 patients with ulcerative colitis and 12 patients with Crohn’s disease) and 31 control subjects. Baseline demographic and clinical data were collected and are synthesized in Table 1.

### 3.2. Hair Concentration of Micronutrients

**Figure 1:**
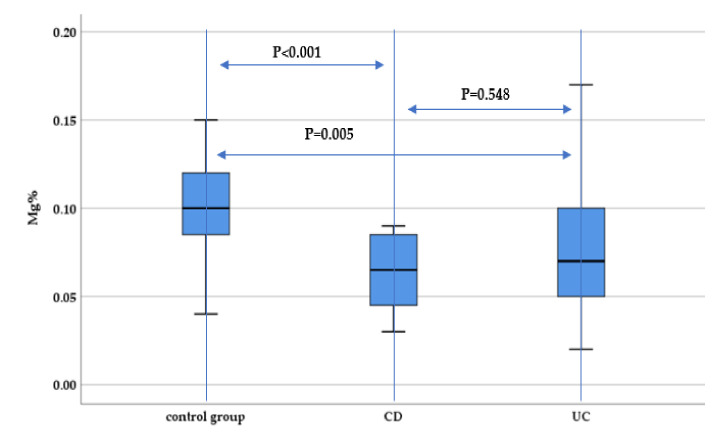
Evaluation of hair magnesium concentration (Mg%) between IBD subtypes (UC and CD) and the control group.

When evaluating the magnesium concentration in IBD patients, there was a statistically significant lower magnesium concentrations both for CD and UC patients compared to controls (*p* < 0.001 and *p* = 0.005, respectively), with no significant differences between UC and CD patients (*p* = 0.548) (Figure 1).

**Figure 2:**
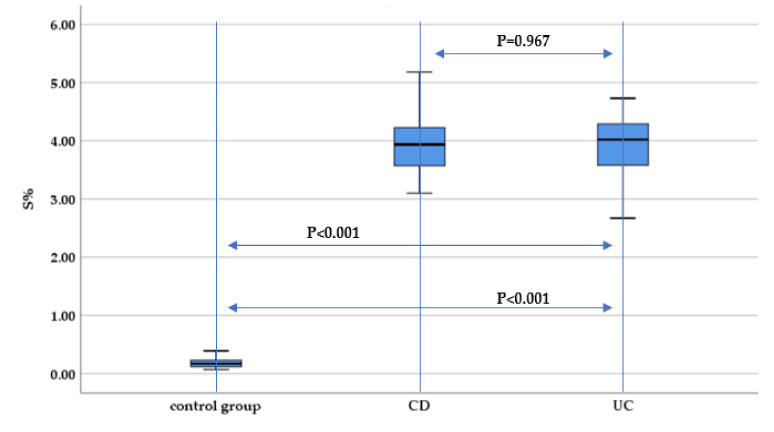
Evaluation of hair sulfur concentration (S%) between IBD subtypes (UC and CD) and the control group.

Measuring sulfur concentration, we identified a significantly increased hair sulfur concentration both in UC and CD patients compared to the control group (*p* < 0.001), without significant differences between IBD subtypes (*p* = 0.967) (Figure 2).

**Figure 3:**
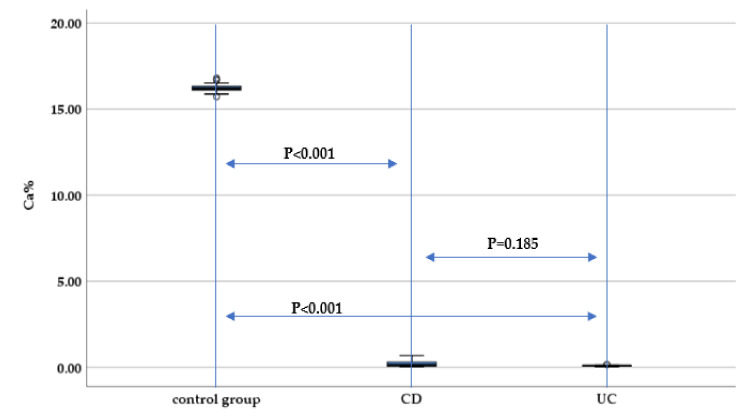
Evaluation of hair calcium concentration (Ca%) between IBD subtypes (UC and CD) and the control group.

Measurement of hair calcium concentration revealed statistically significant lower concentrations both for UC and CD patients compared to the control group (*p* < 0.001). There were no significant differences for hair calcium concentration between UC and CD patients (Figure 3).

**Figure 4:**
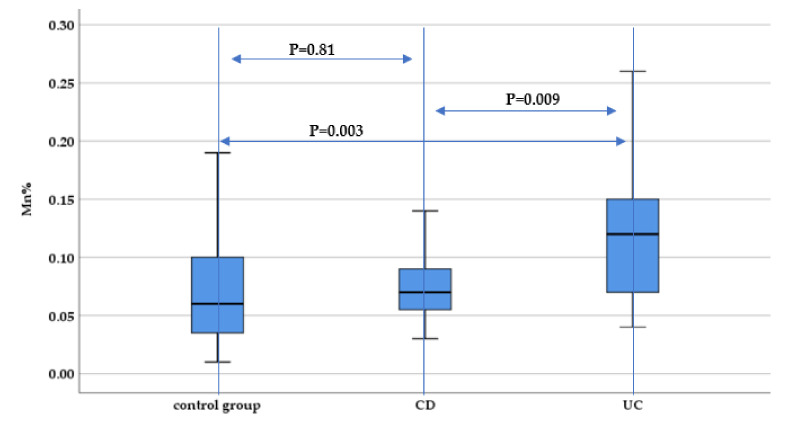
Evaluation of hair manganese concentration (Mn%) between IBD subtypes (UC and CD) and the control group.

Hair manganese concentration was elevated in both the UC and CD group, compared to the control group, reaching statistically significant differences when comparing UC patients with the control group (*p* = 0.003), and between the UC and CD group (*p* = 0.009), but without statistical significance when comparing CD patients and the control group (*p* = 0.81) (Figure 4).

**Figure 5:**
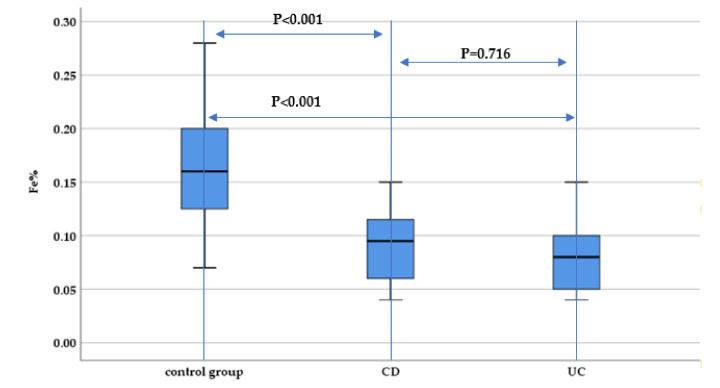
Evaluation of hair iron concentration (Fe%) between IBD subtypes (UC and CD) and the control group.

The hair iron concentration was statistically significantly lower both in CD and UC patients compared to the control group (*p* < 0.001). However, no statistically significant difference between UC and CD patients regarding hair iron concentration was identified (*p* = 0.716) (Figure 5).

**Figure 6:**
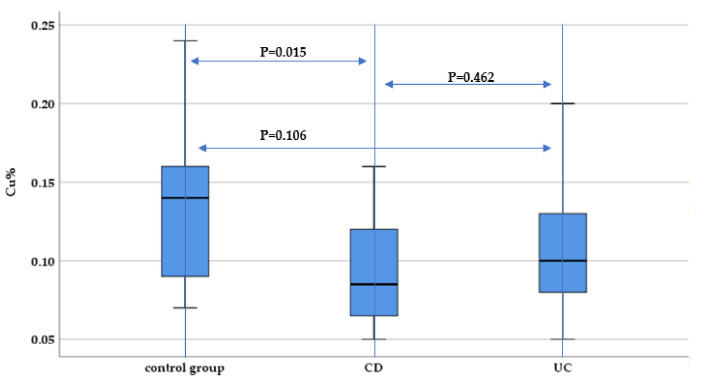
Evaluation of hair copper concentration (Cu%) between IBD subtypes (UC and CD) and the control group.

When evaluating copper hair concentration, we identified significantly lower concentrations among CD patients compared to the control group (*p* = 0.015). There was no statistically significant difference between UC and CD patients regarding hair copper concentration (*p* = 0.462) (Figure 6).

**Figure 7:**
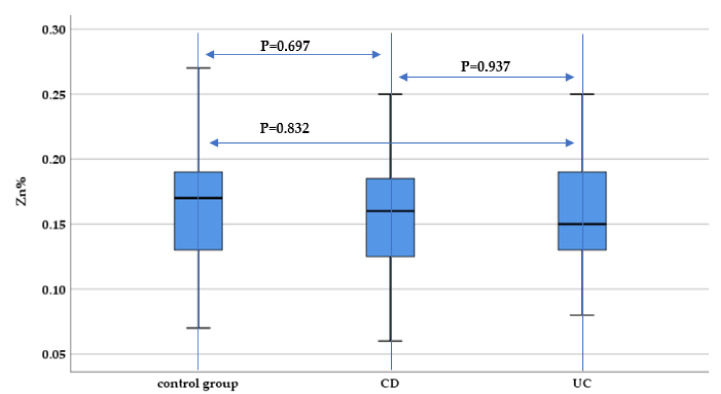
Evaluation of hair zinc concentration (Zn%) between IBD subtypes (UC and CD) and the control group.

As far as zinc concentration in hair is concerned, there were no statistically significant differences between IBD patients and the control group (*p* = 0.697 and *p* = 0.832), although a slightly lower median hair zinc concentration was identified for UC and CD patients compared to the control group (Figure 7).

**Figure 8:**
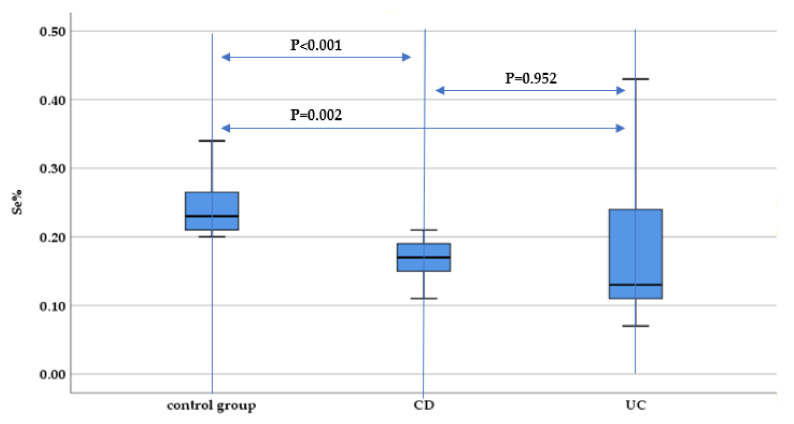
Evaluation of hair selenium concentration (Se%) between IBD subtypes (UC and CD) and the control group.

Hair selenium concentration was decreased both among UC and CD patients compared to the control group (*p* = 0.002 and *p* < 0.001, respectively). However, there was no statistically significant difference between selenium hair concentration between UC and CD patients (*p* = 0.952) (Figure 8).

### 3.3. Evaluating the Potential Correlations between Disease Activity and Mineral and Trace Elements Status

We evaluated the correlation between the hair concentration of the studied minerals and trace elements and disease activity reflected through the Mayo score for UC patients and the CDAI score for CD patients. We identified a statistically significant negative correlation between hair calcium concentration and disease activity for CD patients, but without reaching the level of statistical significance for UC patients. Moreover, there was a strong positive correlation between hair sulfur concentration and disease activity among CD patients, but not for UC patients (Table 2).

The correlations evaluated between the markers of systemic inflammation and active disease (CRP, CRP/albumin, fibrinogen, neutrophil-to-lymphocyte ration and fibrinogen) and mineral and trace elements concentration in hair did not reach the level of statistical significance (Table 3).

When studying the correlation between each type of micronutrients and BMI, we did not find any statistically significant correlation between the hair concentration of any of the studied micronutrients and BMI in the study group nor in the control group (Table 4).

## 4. Discussion

The alteration of nutritional factors among IBD patients might be perceived at a first glance as related to a macronutrient deficit, considering the importance of the altered protein status, especially hypoalbuminemia on IBD evolution and complications [10,11]. Beyond protein malnutrition, micronutrient deficits, including trace elements, should also be considered, since this issue might affect up to half of the patients with IBD [12]. Although this aspect might be of higher impact at the beginning of the disease course, especially for patients with longer diagnostic delay, various nutritional deficiencies might appear, persist or worsen throughout the disease course, due to poor intake and impaired absorption [4].

The most frequently used method for evaluating trace elements is measuring serum concentration, as it is an accessible method [13,14]. Since there is prior evidence that inflammation may alter the serum micronutrient concentrations, the use of serum levels is limited in reflecting the body nutrient status in chronic inflammatory diseases such as IBD [4,15,16]. Considering these aspects, an alternative to be considered for evaluating trace elements status is measuring the hair concentration. The approach of the measurement of multiple elements in the scalp hair is being applied to several diseases [17], ranging from diabetes [18] to autoimmune disorders. There is evidence for the usefulness of trace element evaluation in patients with psoriasis [19] and alopecia areata [9]. There have also been some isolated studies using hair samples for measuring trace elements concentration in patients in specific settings [20]. Taking into account the previously mentioned aspects, we opted for mineral and trace elements evaluation by measuring hair concentration using SEM and EDX.

One patient characteristic which might influence the hair concentration of various elements is age. Among the IBD patients included in our study, the UC patients had a higher age (on average almost 10 years older), compared to the control group and CD patients, although the statistical significance of this difference reached borderline significance. The absence of a significant age difference between CD patients and the control group, together with registering significantly lower hair calcium concentration among CD patients compared to the control group, might be an indicator of calcium reduction in young CD patients. Regarding both calcium and magnesium concentrations, one study analyzing the age dependence of various elements concentrations highlighted higher calcium and magnesium concentrations in younger subjects (those under 25 years old). Consequently, the authors stated that there may be an age dependence of the concentration of these trace elements, but this is difficult to demonstrate regarding scalp hair concentration if the number of samples collected per age group is not large enough [21]. As far as the concentration of other studied elements is concerned, the same study shows that sulfur, zinc and copper concentrations do not significantly vary with age [21].

We identified lower hair concentrations for several minerals and trace elements between IBD patients and healthy controls, namely lower iron, magnesium, calcium and selenium, with statistically significant differences between patients and the control group. Our results are consistent with a previous study that included pediatric IBD patients and measured both serum and hair concentration of trace elements [4]. In this study, there were no statistically significant differences in the hair concentration of these four elements between patients with UC compared to CD patients. While the hair iron deficiency might not be of clinical significance, considering that extensive research has already been done related to the importance of the righteous management of low serum iron levels and the presence of anemia in IBD patients, with practical guidelines established [22,23], there is still a need for better understanding of other micronutrients’ status and management.

The magnesium deficit is of importance in clinical practice and requires prompt diagnosis and management, considering its pivotal role in cell signaling, genomic stability and DNA repair processes [24,25,26], but also due to its influence on other minerals such as the transport of calcium [26]. Beyond the typical clinical picture of low magnesium levels, such as muscle cramps, arrhythmia and impaired tissue repair, the importance of magnesium deficit is also reflected in accentuating depression and enhancing fatigue among IBD patients [27]. Therefore, magnesium supplementation could contribute to improving IBD evolution for patients with a deficit of this mineral, but further evidence is needed in order to generate specific dosing, time of supplementation and optimum monitoring of magnesium status in IBD patients.

Another mineral which was deficient among the studied IBD group was calcium, which was also negatively correlated with disease activity for CD patients. These findings suggest the presence of a calcium deficit in CD patients, especially during disease flares. Calcium deficiency is pathophysiologically well-founded, considering that calcium is mainly absorbed in the ileum [28] and that almost 40% of the CD patients included in our study did have ileal involvement. This underlines the importance of monitoring calcium status in IBD patients, especially among CD patients and particularly during disease flare, when the presence of ileal inflammation impairs calcium absorption, which will mainly take place through transcelullar active transport [29] in this setting. Beyond the impairment of calcium absorption and the negative influence on vitamin D status, data are less extensive regarding the role of acute on chronic inflammation over the calcium homeostasis at both renal and intestinal level in IBD patients. One study using a mouse model of CD ileitis with a deletion in the tumor necrosis factor (TNF) AU-rich elements (ARE), characterized by elevated TNF-alpha levels, identified that in spite of maintaining a normal calcium level, ileitis with increased TNF-alpha expression led to a disturbed calcium homeostasis. This was characterized by reduced duodenal and renal calcium transporters, diminished 1,25(OH)2D3 levels and increased bone resorption associated with profound bone abnormalities [30]. These findings might suggest that a normal serum calcium level does not necessarily reflect normal calcium metabolism in IBD patients; therefore, measuring the hair calcium concentration might represent an option for better reflecting calcium status in this setting, in order to ensure an optimized monitoring.

Selenium represents an important trace element in modulating the anti-inflammatory and antioxidant response, considering that it is part of glutathione-peroxidase and thioredoxin reductase. Moreover, the selenium deficiency can negatively influence DNA repair and cell cycle regulation [31,32,33]. We identified statistically significant lower selenium concentrations for both UC and CD patients compared to the control group, results which are in accordance with other study evaluating selenium deficits in IBD patients compared to healthy controls, referring to serum concentration [34]. However, these results are opposed to those reported by Cho JM and Yang HR, who did not identify statistically significant lower selenium concentration for IBD patients compared to healthy controls when evaluating serum level nor when evaluating hair concentration of this trace element [4]. Since there are conflicting results for evaluating selenium status in IBD patients, considering the various methods used to determine its concentration but also considering the multiple potential causes of variation of its concentration, it is difficult to underline the optimum approach for evaluating selenium status in this patient category.

The common encounter of zinc deficiency reported in patients with IBD (15 to 40% of the patient population) [35] is not surprising, considering the chronic diarrhea and malabsorption during disease flares and the key role zinc plays in the maintenance of intestinal barrier integrity [36]. On the other hand, zinc deficiency has also been reported for patients in remission, reaching up to one-third of patients [37,38]. While zinc is generally thought to play an important role in fighting oxidative stress, it is also important for keratinocyte proliferation in hair [39]. These roles could explain the presence of zinc deficiency among IBD patients with long standing disease course. In addition, a study including IBD patients highlighted that low serum zinc concentration might be associated with poor disease outcome, reflected by increased rate of complication, increased need for hospitalization and surgery [40].

However, in our study, we did not identify statistically significant differences between hair zinc concentration between IBD patients and the control group. This particular finding, in spite of the important involvement of zinc deficiency in oxidative stress and intestinal permeability, might partly be related to the low number of patients included in the study. Our results are consistent with the ones reported by Cho JM and Yang HR, who did not identify significant differences between hair zinc concentration between IBD and controls, although when evaluating serum concentrations of this trace elements, the differences were significant and pointed towards a zinc deficit among IBD patients [4]. Corroborating these findings, we might presume that there is a relative serum zinc deficiency related to the inflammatory process, without affecting the zinc deposits, such as the zinc incorporated in the hair, and therefore, potentially overestimating the degree of zinc deficiency in this patient category. Still, lower serum zinc level might be useful in predicting an increase of disease activity, considering that one recent study investigating micronutrient deficiency on CD outcome identified a significant association between low serum zinc concentration and shorter time until disease flare for patients who were in remission at the moment of inclusion in the study [41]. Consequently, there is a need for further prospective studies evaluating both serum and hair zinc concentration in order to identify the best way to integrate these findings in the clinical setting.

Hair copper concentration was lower for IBD patients compared to healthy controls in our study, but the difference reached the level of statistical significance only for CD patients, compared to the control group. This finding might be explained by the impaired copper absorption among CD patients with ileal involvement. Moreover, taking into account previous research highlighting that copper incorporated from endogenous sources is securely bound within the hair, without being lost upon exposure to the environment [42], we can assume that low copper concentration in the hair of CD patients might reflect a true deficit in this context.

With regard to manganese status, we identified a statistically significant higher concentration of this trace element among UC patients, but not in CD patients, compared to the control group. There was no statistically significant correlation between hair manganese concentration and parameters reflecting inflammation and disease activity scores, neither for UC nor for CD patients in the study group. These findings might be due to the low number of patients included and further studies involving larger patient group could offer better insight regarding the status of manganese among IBD patients. More data on manganese status among IBD patients could be useful, considering that currently, manganese is a less studied trace element for this patient category, although it has an important antioxidant role, as part of manganese-superoxide dismutase and also as a cofactor of xantinoxidase, arginase, pyruvate decarboxylase and glutaminsynthetase [43]. A study using a mouse model highlighted that manganese deficiency exacerbates intestinal injury and inflammation, promoting increased intestinal permeability, through alteration of tight junction expression [44]. These data, together with the identification of manganese deficiency in a study including pediatric patients recently diagnosed with IBD [4], support the opportunity to further investigate the role of this trace element in the pathogenesis of IBD. Furthermore, it is also important to find the optimum method to assess patients’ nutritional manganese status, especially for making recommendations regarding dietary habits to those at a high risk of IBD. Currently, no formal recommended dietary allowance for manganese is available for IBD patients, where the need for this trace element might be higher than in healthy individuals, for whom the Institute of Medicine’s Dietary Reference Intake cites ~2 mg manganese per day as adequate for adults [45].

Another chemical element for which we found increased hair concentration in IBD patients compared to healthy control is sulfur. Since the main hair structural component is keratin, which includes disulfide bonds [46], we can presume a change in hair incorporation of sulfur among IBD patients. On the other hand, increased sulfur concentration in the hair of IBD patients might be attributed to inflammation, since in the studied group, we identified a statistically significant correlation between the sulfur concentration and CD activity. Moreover, increased hair sulfur concentration can be related to the consequences of oxidative stress, namely the increased expression of metallothioneins, a type of cysteine-rich Zn/Cu-binding proteins, which is amplified at the keratinocyte level [9,47].

The oxidative stress in the context of IBD might be related to the deficit of copper we identified among IBD patients compared to the control group, considering that copper is part of the copper-zinc superoxide-dismutase. This activity of this enzyme has been previously reported to be reduced in IBD patients, although the mechanism of this decreased activity is very intricate and involves both chronic inflammation and zinc deficit [48,49]. As far as other reported data on copper status in IBD patients are concerned, there are conflicting data on comparing copper concentration in IBD patients to healthy controls [50,51].

The investigation we performed on mineral and trace elements concentration in the hair of IBD patients is among the few currently available and might bring valuable data, especially considering the extended panel of elements evaluated through EDX, including data on copper and manganese, which have been less investigated among IBD patients. The hair evaluation of micronutrient status has become of increasing interest, especially for chronic, inflammatory diseases, since the measurement is not directly influenced by the value of serum inflammatory markers or by recently ingested food and is completely non-invasive to sample. Moreover, hair concentration of various micronutrients could better reflect their status for a longer period than the serum evaluation, which offers information about the recent status of the elements from the moment of sampling. However, there are several factors which might be incriminated in influencing the micronutrient status and hair composition, such as diet, nutritional status or the time of disease evolution. To avoid a significant influence of the general nutritional status on micronutrient concentration, we have studied the correlation between BMI and the concentration of the studied micronutrients and found no statistically significant correlation in the control group nor in the study group. Since the groups did not differ with a statistical significance with regard to BMI and this parameter was within comparable ranges among the studied groups, it is difficult to shape further conclusions with regard to the influence of BMI on the status of hair micronutrients.

Furthermore, our patient group was very heterogeneous regarding time from disease diagnosis, ranging from patients just recently diagnosed to patients with long standing disease; additionally, considering the low number of patients included, we did not analyze the correlation between the time of disease course and micronutrient status. This lack of analysis represents a limitation of our study and it would be of interest for future studies involving a higher number of patients, since persistent inflammation, longer disease course and presence of ileal involvement reduce the absorption of various nutrients, which could negatively influence the micronutrient status. There are also some other limitations to the study performed, related to the unavailability of concomitantly measuring the serum concentration of micronutrients. The corroboration of data from more patients, together with concomitant evaluation of hair and serum concentration, would bring a better reflection of the status of these minerals and trace elements and could contribute to optimally assessing their concentration, and therefore, enable shaping clear recommendations for specific supplementation where applicable. Furthermore, adding a systematic approach of the patients’ diet could offer insight into identifying which deficits are also influenced by dietary restrictions and concomitantly assessing fecal calprotectin could contribute to better understanding the involvement of the intestinal inflammation in the micronutrient deficiency. To a certain extent, the concomitant evaluation of vitamin D status could also be of use, especially considering its prevalent deficit among IBD patients, even for quiescent disease and its influence on the phosphocalcic metabolism [37].

## 5. Conclusions

In the performed study, we identified significantly lower hair concentrations for iron, magnesium, calcium and selenium among IBD patients (both for UC and CD) compared to healthy controls, without significant differences between UC and CD patients. The presence of some of these deficits was correlated with disease activity and inflammatory markers. Sulfur hair concentration was significantly higher among both UC and CD patients compared to healthy controls. Measuring the hair concentration of various minerals and trace elements might represent a reliable method for evaluating their status among IBD patients and contribute to a better integration of micronutrient supplementation in the disease management.

## Figures and Tables

**Table 1:** Patient characteristics.

Patient Characteristics	Study Group *n* = 37		Control Group *n* = 31	*p*-Value
	UC (*n* = 25)	CD (*n* = 12)		
Age, median (Q25; Q75)	43.5 (30; 59.5)		32 (29; 42)	0.05 §
	46 (32.5; 65.5)	33 (27.5; 44.5)		
Sex (M/F), *n* (%)	19/18 (51.4/48.6)		16/15 (51.6/48.4)	0.981 Ϯ
	13/12 (52/48)	6/6 (50/50)		
Urban vs. rural area (U/R), *n* (%)	29/8 (78.4/21.6)		28/3 (90.3/9.7)	0.286 Ϯ
	20/5 (80/20)	9/3 (75/25)		
BMI (average ± SD)	21.97 ± 1.5		23.08 ± 2.2	0.259 #
	22.61 ± 1.95	21.97 ± 1.5		
Disease activity score	Mayo score 3 (1; 7)	CDAI score 146.5 (52.5; 276.5)		
Active disease, *n* (%)	22 (59.5%)		NA	NA
	16 (64% *)	6 (50% *)	NA	0.65 Ϯ
Disease location *	Proctitis 6 (24%) Left-sided colitis 13 (52%) Pancolitis 6 (24%)	Ileum (L1) 2 (16.6%) Colonic (L2) 7 (58.3%) Ileocolic (L3) 3 (25%)	NA	
Treatment followed *	aminosalicylates 18 (72%) azathioprine 1 (4%) biological therapy 4 (16%) first diagnosis-no prior treatment 2 (8%)	azathioprine 6 (50%) biological therapy 4 (33.3%) first diagnosis-no prior treatment 2 (16.7%)	NA	

* percentage according to disease subtype, § Kruskal–Wallis test, Ϯ Chi-square test, # one-way ANOVA. UC-ulcerative colitis, CD-Crohn’s disease, BMI-body mass index, CDAI-Crohn’s disease activity index, SD-standard deviation, NA-not applicable.

**Table 2:** Evaluating the correlations between mineral and trace elements and disease activity.

Evaluated Element	CD Disease Activity		UC Disease Activity	
	Correlation Coefficient	*p*-Value	Correlation Coefficient	*p*-Value
Mg% ^╥^	−0.147	0.649	0.186	0.375
S% ^	0.585	0.046 *	0.112	0.594
Ca% ^╥^	−0.772	0.003 *	−0.058	0.782
Mn% ^╥^	−0.269	0.398	0.133	0.525
Fet% ^╥^	0.315	0.319	0.006	0.978
Cu% ^╥^	−0.024	0.940	0.052	0.804
Zn% ^	0.696	0.126	0.009	0.966
Se% ^╥^	0.269	0.398	−0.006	0.978

^ Pearson correlation test, ^╥^ Spearman correlation test; * marked results are significant for *p* < 0.05.

**Table 3:** Evaluating the correlation between hair micronutrient concentration and inflammatory markers for IBD patients.

Evaluated Element	CRP		CRP/ALB		NLR		Fibrinogen	
	Correlation Coefficient	*p*-Value	Correlation Coefficient	*p*-Value	Correlation Coefficient	*p*-Value	Correlation Coefficient	*p*-Value
Mg% ^╥^	−0.284	0.088	−0.257	0.125	−0.261	0.119	−0.288	0.094
S% ^	0.135	0.425	0.145	0.391	0.171	0.311	0.206	0.236
Ca% ^╥^	−0.297	0.074	−0.301	0.07	−0.266	0.112	−0.119	0.497
Mn% ^╥^	0.185	0.274	0.161	0.342	0.112	0.511	−0.255	0.139
Fe% ^╥^	−0.133	0.432	−0.122	0.474	−0.003	0.987	−0.154	0.379
Cu% ^╥^	−0.110	0.516	−0.130	0.442	0.016	0.987	−0.279	0.105
Zn% ^	−0.061	0.722	−0.06	0.725	0.063	0.713	−0.094	0.589
Se% ^╥^	0.144	0.396	−0.043	0.799	0.059	0.728	0.189	0.277

^ Pearson correlation test, ^╥^ Spearman correlation test, CRP/ALB = CRP-to-albumin ratio, NLR = neutrophil-to-lymphocyte ratio.

**Table 4:** Evaluating the correlation between the type of micronutrients and BMI.

Evaluated Element	UC Patients		CD Patients		Control Group	
	Correlation Coefficient	*p*-Value	Correlation Coefficient	*p*-Value	Correlation Coefficient	*p*-Value
Mg% ^╥^	−0.002	0.993	−0.099	0.759	0.143	0.442
S% ^	−0.01	0.962	0.333	0.290	−0.2	0.917
Ca% ^╥^	0.088	0.675	−0.147	0.649	−0.083	0.657
Mn% ^╥^	0.113	0.592	0.563	0.057	−0.006	0.976
Fe% ^╥^	0.023	0.911	−0.011	0.974	−0.268	0.144
Cu% ^╥^	0.091	0.665	0.204	0.526	0.225	0.220
Zn% ^	−0.087	0.678	0.409	0.187	−0.198	0.285
Se% ^╥^	−0.272	0.189	0.309	0.329	0.077	0.680

^ Pearson correlation test, ^╥^ Spearman correlation test.

## Data Availability

The datasets generated during the current study are available from the corresponding author on reasonable request.
